# Progress in Medicine

**Published:** 1895-02-09

**Authors:** 


### Progress in Medicine.

### DISEASES OF THE INTESTINES.

**Fæcal Concretions.**—Two somewhat similar cases are recorded by Dr. George Middleton[1] and Dr. Ott[2], in which large fæcal masses gave rise to serious symptoms in women. In Dr. Middleton's case, a tumour was felt in the neighbourhood of the umbilicus in a weak, emaciated woman, æt. 32. It was indefinite in outline, doughy, and somewhat movable. She had suffered from vomiting for nine weeks. The bowels were habitually constipated. After being in the infirmary a few days, the temperature rose to 104 deg. The vomiting continued, but the bowels were open two or three times daily. Death occurred about twelve weeks from the time of onset of the illness. At the post-mortem the colon was found full of hard fæcal masses. Death was attributed to absorption of poisons from the intestinal canal. Dr. Ott's case was in a woman aged 56. There was abdominal pain, diarrhœa, and fever. No mass could be felt on abdominal examination; but after a time the patient felt a hard body move towards the rectum. This hard body proved to be a fæcal concretion, which was broken up and removed, after which all bad symptoms disappeared.

**Intestinal Putrefaction.**—Dr. Herter, of New York,[3] draws numerous conclusions from his observations upon the results of intestinal putrefaction. Amongst these, he states that indigo blue in the urine is not necessarily proportional to the total quantity of aromatic sulphates present, but that the regular occurrence of more than a trace of indigo blue is to be regarded as pathological. He considers that some of the early epileptic seizures of children may be due to the absorption of products of decomposition from the intestines, and that treatment directed to the correction of putrefactive excess may cure some cases of this kind.

**Milk Diet and Intestinal Decomposition.**—Dr. J. A. Weisner, of Chicago, gives the result of some experiments upon the cause of the reduction of the aromatic sulphates in the urine when the diet is milk. Dr. Weisner administered koumiss to typhoid fever patients, and found the aromatic sulphates very greatly reduced, sometimes as much as 85 per cent. Lactic acid is present in koumiss, and Dr. Weisner considers this product to be the cause of the antiseptic action of milk. The effect of diet upon the urine of a healthy person was afterwards tested. The amount of aromatic sulphates was estimated when ordinary diet was taken in association with salol or turpentine. They were again estimated when the diet was buttermilk without being accompanied by drugs. The aromatic sulphates were much smaller in amount with the milk diet than with ordinary food and intestinal antiseptics. In conclusion, Dr. Weisner states that he considers that koumiss should be given in cases of intestinal putrefaction.

**Pseudo-diverticulum of the Duodenum.**—Pilcher[4] records a case of pseudo-diverticulum of the ileum in a boy aged seventeen, whose health had been good up till twelve weeks before admission. He then suffered from fever and abdominal tenderness. An indefinite tumour could be felt below the umbilicus. At the operation a fluctuating, heart-shaped tumour was found situated behind the peritoneum, stretching from the transverse colon above to the brim of the pelvis below. It was opened, and brown puriform fluid escaped. The boy died on the third day of peritonitis. The tumour was found to be a thick-walled sac, capable of holding six ounces of fluid, into which the stomach, jejunum, and bile duct opened. The sac was lined with inflammatory connective tissue, with the exception of a narrow strip where mucous membrane was present.

**Rheumatic Perityphlitis.**—Dr. Burney Yeo[5] records an interesting case of a girl aged eighteen admitted with signs of appendicitis, who three days after admission was attacked with arthritis of several joints. Salicylate of soda was given which allayed the joint inflammation. As soon as the drug was stopped the symptoms of perityphlitis increased in severity, and Dr. Burney Yeo considered the advisability of calling in a surgeon. He decided, however, to again administer the salicylate of soda. In twenty-four hours the temperature fell to normal and the symptoms of perityphlitis rapidly disappeared.

Dr. Alexander Haig[6] commented upon this case and pointed out that he had recorded several similar cases under the name of gout of the intestines, and had also noticed that they clear up under the administration of salicylate of soda.

**Appendicitis Obliterans.**—Dr. Senn[7] states that appendicitis obliterans is a comparatively frequent form of relapsing inflammation of the vermiform appendix. The various structures of the appendix gradually disappear and the entire lumen becomes obliterated. The changes in the appendix may either follow superficial ulceration or be interstital from the commencement following lymphatic infection. The obliteration may commence at either the proximal or the distal end, or may affect several points at the same time. Obliteration of the proximal end may give rise to retention of septic material which finds an outlet through the lymphatics giving rise to suppurative or non-suppurative lymphangitis. Circumscribed plastic peritonitis is an almost constant concomitant of this form of peritonitis and hastens the obliteration. Complete obliteration of the lumen results in a permanent cure. Dr. Senn, however, remarks that since prolonged suffering attends such spontaneous cure, and since the process of cure is attended with various dangers, a radical operation is indicated.

**Peritonitis.**—Treves[8] points out that death in peritonitis is caused by the absorption of toxines, and that peritonitis resembles other inflammations in being an evidence of attempts on the part of the serous membrane to protect the individual. He also lays stress upon the fact that the varieties of peritonitis are due to infective organisms, stating that although it has been supposed that mechanical, thermal, or chemical irritants can produce peritonitis, there is not sufficient evidence to prove that such irritants acting alone can give rise to the inflammation. The organism present in cases of peritonitis due to diseases of the intestine, such as appendicitis, or typhoid fever, is the bacterium coli commune. In peritonitis due to infection from without streptococci are found.

Mr. Treves gives valuable suggestions in connection with treatment. He thinks that the rigid prohibition of food and drink by the mouth is often needlessly cruel. The patient longs for a drink of water, and although the indulgence causes vomiting, he is greatly relieved. On the other hand, the perpetual sucking of ice fills the stomach with cold water, and adds to the depression of the patient. The right course appears to lie between the extremes of exclusion of liquor and the perpetual ice-taking. With regard to opium, it ought not to be given as a routine method of treatment. The indication for its use is actual pain. The value of aperients in certain cases is also alluded to. The beneficial effect of evacuation of the intestines in cases of inflammation in the abdominal cavity is illustrated by the fact that cases of appendicitis associated with diarrhœa have a lower mortality than those in which constipation is present. It is in cases of appendicitis seen early that Mr. Treves mainly recommends aperients. Operative interference, as would be expected, is successful where the collections of pus or of sero-purulent fluid are localised, but in generalised peritonitis of acute nature results have not been very encouraging.

**Tubercular Peritonitis.**—Mr. Treves[9] gives a valuable summary of the leading features of tubercular peritonitis. He refers to the clinical and pathological varieties of the disease. The onset is sometimes acute with fever and abdominal pain, but much more often insidious; the abdomen swells, and there is gradual deterioration of the patient's health. Mr. Treves rapidly outlines the leading physical signs presented by the enlarged abdomen. "The abdomen becomes considerably swollen, and may exhibit almost every possible gradation and combination of tympanites and dulness of percussion. More or less exudation is usual, and may be general or may be confined to one or more districts; the intestines may be matted together into a cyst-like mass; or the omentum may be rolled into a solid ball, not to be readily distinguished from a substantial growth. Adhesions may lead to a multitude of curious phenomena, discoverable on palpation. Here may be an area of resistance, and there a void; here all the manifestations of coils of intestines bound down by bands and fillets, and there signs of bowel floating free in a thin fluid. There may be the phenomena of abscess on one side of the abdomen, and the physical signs of ascites on the other." In order that it may be most clearly apprehended what cases are suited for surgical treatment, Mr. Treves quotes Aldibert's classification of the disease into (1) ascitic, (2) fibrous, and (3) ulcerous varieties. The ascitic form may be acute, sub-acute, or chronic. No adhesions are present, but there is marked ascites, and grey granulations are scattered over the serous membrane. In the fibrous form there is considerable development of fibrous tissue, but the ascites is slight. Two varieties of that form are recognised—the dry and the adhesive. In the dry form large tubercles are found scattered over the peritoneum, but there are no adhesions, and no ascites is present. In the adhesive variety the coils of intestine are matted together by bands. In the ulcerous form the intestines are also matted together, but mixed up with adhesions are masses of caseous material and small cystic collections of fluid or pus. Here and there, too. adherent coils may have ulcerated into one another, As we should expect, the operation of opening the abdominal cavity has been most successful in chronic cases, and especially where the fluid has been encysted. Treves quotes from Aldibert's list of 300 cases of laparotomy for tubercular peritonitis. In 101 cases of the ascitic variety operated upon, there were 31 deaths and 35·4 per cent. were considered complete recoveries. Of 149 cases of the fibrous form the percentage of cures is put down at 76·9, of which two-fifths can be considered complete recoveries. Of the ulcerous form 59 per cent. are called cures, and of these one-fourth complete recoveries. The best results appear to have followed simple incision without either flushing or drainage, yet it has been proved that repeated puncture is valueless in the great majority of cases. At present no definite explanation can be given of the beneficial effects of laparotomy. The admission of light, the entrance of air, removal of toxines, and alteration of abdominal pressure have all been considered, but it is not clear how any of them can start the process of cure. Nolen,[10] however, acting upon the hypothesis that air is the therapeutic agent, has introduced it into the abdominal cavity through a needle. Two of three cases treated by this method were successful.

**Chylous Ascitis.**—Weis[11] and Bargebuhr[12] record cases of chylous ascitis associated with deep-seated malignant growth involving the abdominal lymphatics. Microscopical examination of the fluid showed leucocytes of various sizes undergoing fatty degeneration.

[1] Glasgow Med. Journal, May 1894. [2] Prager Med. Woch., 1894. [3] Med. Week, Feb., 2nd, 1894. [4] Annals of Surgery. July, 1894; Brit. Med., August 11th, 1894. [5] Brit. Med. Journal, June 16th, 1894. [6] Brit. Med. Journal, June 30th, 1894. [7] Internat. Med. Magazine, June 1894. [8] Leitomian Lectures, Brit. Med. Journal, Feb. 3rd, 1894, [9] Annals of Surgery, May, 1894. [10] Berlin Klin Wochenschrift, 1893; Amer. Journ. Med. Sciences, Mar., 1894. [11] Centralblatt Med., July 21st, 1894; Brit. Med. Journal, August 11th, 1894. [12] Deutches Archiv. für Klin. Med., 1893; Quarterly Med. Journal, April 1894.

